# Telomere Replication: Solving Multiple End Replication Problems

**DOI:** 10.3389/fcell.2021.668171

**Published:** 2021-04-01

**Authors:** Erin Bonnell, Emeline Pasquier, Raymund J. Wellinger

**Affiliations:** Department of Microbiology and Infectious Diseases, Faculty of Medicine and Health Sciences, Cancer Research Pavilion, Université de Sherbrooke, Sherbrooke, QC, Canada

**Keywords:** telomeres, telomeric chromatin, DNA replication, genome stability, replication fork stability

## Abstract

Eukaryotic genomes are highly complex and divided into linear chromosomes that require end protection from unwarranted fusions, recombination, and degradation in order to maintain genomic stability. This is accomplished through the conserved specialized nucleoprotein structure of telomeres. Due to the repetitive nature of telomeric DNA, and the unusual terminal structure, namely a protruding single stranded 3′ DNA end, completing telomeric DNA replication in a timely and efficient manner is a challenge. For example, the end replication problem causes a progressive shortening of telomeric DNA at each round of DNA replication, thus telomeres eventually lose their protective capacity. This phenomenon is counteracted by the recruitment and the activation at telomeres of the specialized reverse transcriptase telomerase. Despite the importance of telomerase in providing a mechanism for complete replication of telomeric ends, the majority of telomere replication is in fact carried out by the conventional DNA replication machinery. There is significant evidence demonstrating that progression of replication forks is hampered at chromosomal ends due to telomeric sequences prone to form secondary structures, tightly DNA-bound proteins, and the heterochromatic nature of telomeres. The telomeric loop (t-loop) formed by invasion of the 3′-end into telomeric duplex sequences may also impede the passage of replication fork. Replication fork stalling can lead to fork collapse and DNA breaks, a major cause of genomic instability triggered notably by unwanted repair events. Moreover, at chromosomal ends, unreplicated DNA distal to a stalled fork cannot be rescued by a fork coming from the opposite direction. This highlights the importance of the multiple mechanisms involved in overcoming fork progression obstacles at telomeres. Consequently, numerous factors participate in efficient telomeric DNA duplication by preventing replication fork stalling or promoting the restart of a stalled replication fork at telomeres. In this review, we will discuss difficulties associated with the passage of the replication fork through telomeres in both fission and budding yeasts as well as mammals, highlighting conserved mechanisms implicated in maintaining telomere integrity during replication, thus preserving a stable genome.

## Introduction

Genome stability is maintained by appropriate genome duplication and conservation of chromosomal integrity. In eukaryotes, the ends of linear chromosomes are known as telomeres, and are associated with specific nucleoprotein complexes that are essential in preventing genome instability. Telomere-associated proteins help avoid unwanted events such as chromosomal fusions or chromosomal rearrangements by preventing recognition of telomeres as double-strand breaks (DSBs) [reviewed in (Wellinger and Zakian, 2012; De Lange, 2018)]. With few exceptions, telomeric DNA is comprised of short, repetitive non-coding TG-rich sequences ending in a 3′ G-rich single-stranded overhang. The G-rich nature of the repeats and presence of a 3′-overhang are characteristics of telomeric DNA that are highly evolutionarily conserved in eukaryotes, although there are variations in the repeat sequence and repeat size depending on the organism (Giraud-Panis et al., 2013). Human telomeres are composed of several kilobases (∼5–15) of TTAGGG tandem repeats and 12–400 nucleotides (nt) of 3′ G-rich single-stranded overhang (Makarov et al., 1997; McElligott and Wellinger, 1997; Zhao et al., 2008). *Saccharomyces cerevisiae* telomeres are comprised of 300 ± 75 bp of double stranded heterogeneous TG_1__–__3/_C_1__–__3_A repeats with a 8-15 nt overhang (Wellinger and Zakian, 2012; Soudet et al., 2014). Similar to *S. cerevisiae* in terms of size and heterogeneous nature, *Schizosaccharomyces pombe* telomeres consist of approximately 300 bp of a degenerate repeat sequence with a common motif of TTACAGG, and a consensus sequence of T_1__–__3_ACA_0__–__2_C_0__–__1_G_1__–__8_ (Sugawara, 1988; Liu et al., 2010).

**FIGURE 1:**
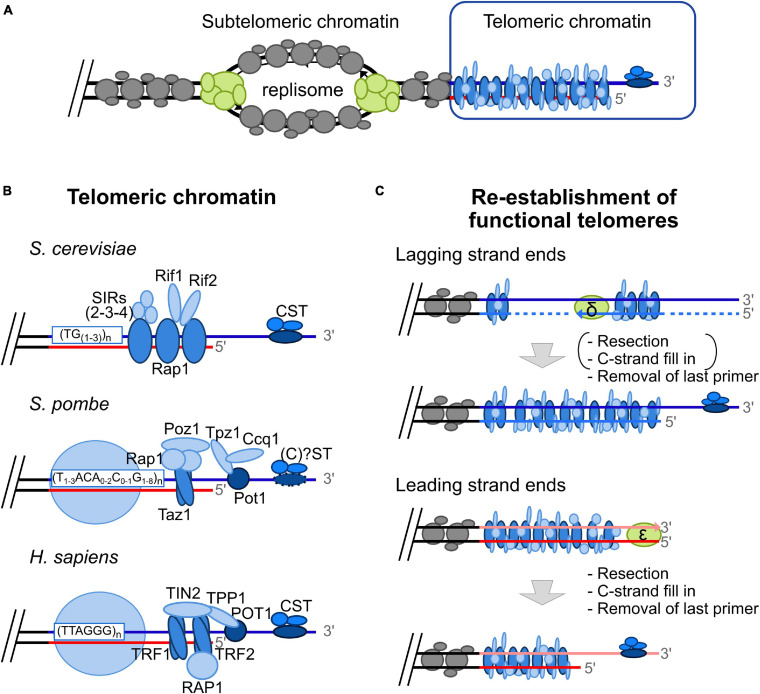
The “Unusual” telomeric chromatin and the “classical” End Replication Problem. **(A)** Replication origins in subtelomeric areas fire in S-phase (humans) or in late S-phase (yeasts). At each fork, the replisome, a protein complex schematized here in green, allows DNA duplication. At the leading strand, DNA is synthesized by DNA polymerase ε in a continuous fashion, whereas at lagging strand, DNA synthesis by DNA polymerase δ occurs in a discontinuous fashion, i.e., in the form of Okazaki fragments. Subtelomeric chromatin is displayed in gray and the unusual telomeric chromatin is represented in blue. **(B)** Telomeric chromatin is unusual due to the binding of specific proteins in a sequence specific manner and lack of classical nucleosomes. Whereas telomeric chromatin in *S. cerevisiae* is devoid of nucleosomes (Wright et al., 1992), histones are present over telomeric repeats in *S. pombe* and humans in a non-canonical fashion (Greenwood et al., 2018). Rap1 recognizes dsDNA budding yeast telomeric repeats [(TG1-3) n] whereas Cdc13p binds the ssDNA telomeric overhang (Wellinger and Zakian, 2012). Telomere-bound Rap1 recruits several proteins such as the SIR complex (Sir2/Sir3/Sir4), and Rif1/Rif2. Cdc13 recruits Stn1 and Ten1, forming the CST complex. In *S. pombe*, Taz1 binds as homodimer on duplex telomeric DNA, whereas Pot1 recognizes single strand telomeric DNA. These two telomere-bound proteins recruit several proteins: Rap1, Poz1, Tpz1, and Ccq1 (Shelterin-like complex) (Moser and Nakamura, 2009). Whereas the homolog of Cdc13 has not been identified in this model organism, Stn1, and Ten1 are known to bind to telomeric ssDNA without forming a complex with the other ssDNA-binding protein Pot1 (Martín et al., 2007). Contrary to the heterogeneous telomeric repeats found in *S. cerevisiae* and *S. pombe*, TTAGGG repeats are found in most vertebrate species, including humans. The Shelterin complex is associated with human telomeric DNA and is comprised of TRF1 and TRF2 bound as homodimers on duplex DNA, POT1 on ssDNA, and associated proteins: RAP1, TIN2 and TPP1 (De Lange, 2005). **(C)** The “classical” End Replication Problem leading to progressive telomere shortening is the consequence of the unusual DNA structure of telomeres, i.e., the constitutive 3′ overhang, that has to be reformed after conventional replication, and the unidirectionality of DNA synthesis by conventional replicative DNA polymerase (from 5′ to 3′). Indeed, the G-rich strand (blue line) is used as DNA template by lagging strand machinery (primase-DNA polymerase α, synthesizing a RNA-DNA primer (dotted line) followed by extension by DNA polymerase δ). Removal of the last primer is expected to be sufficient to reform functional telomeres, at least in yeast. The leading strand machinery (DNA polymerase ε) allows complementary synthesis of the C-rich strand leading to a blunt end. 5′ resection followed by C-strand fill in and removal of the last primer allows re-establishment of functional telomeres. It should be noted that resection and C-strand fill in occur at lagging strand ends in humans [mentioned under parentheses in the scheme; (Wu et al., 2012)].

Like the rest of the genome, telomeres must be accurately duplicated during S-phase to ensure proper cell division. DNA replication is initiated at multiple replication origins in a bidirectional way (Prioleau and MacAlpine, 2016). At each replication fork, the replisome ensures unwinding of parental DNA, followed by DNA synthesis of the complementary strand by conventional DNA polymerases (Figure 1A; Guilliam and Yeeles, 2020). Unwinding of telomeric DNA leads to a temporally restricted disruption of the compacted telomeric chromatin formed by telomere-associated proteins (telomeric chromatin described in Figure 1B). Moreover, without compensatory mechanisms, telomeres shorten progressively at each round of DNA replication, a phenomenon called the End Replication Problem (Wellinger, 2014) (explained in more detail in Figure 1C). In most eukaryotes, this problem is solved by 3′ extension of telomeres by a reverse transcriptase called telomerase, and subsequent fill in by conventional DNA replication machinery (Wellinger, 2014).

This review compares telomeric replication by conventional replicative machineries in humans and two lower eukaryotic model organisms, budding and fission yeasts. We first focus on difficulties encountered by the replisome in reaching the chromosomal ends, followed by a description of possible outcomes of interrupted “conventional” telomeric replication and the main pathways involved in proper telomere replication completion.

## Telomeric DNA Replication by the Conventional Replication Machinery

### Difficulties Associated With Replication Fork Passage Through Chromatinized Telomeres

**FIGURE 2:**
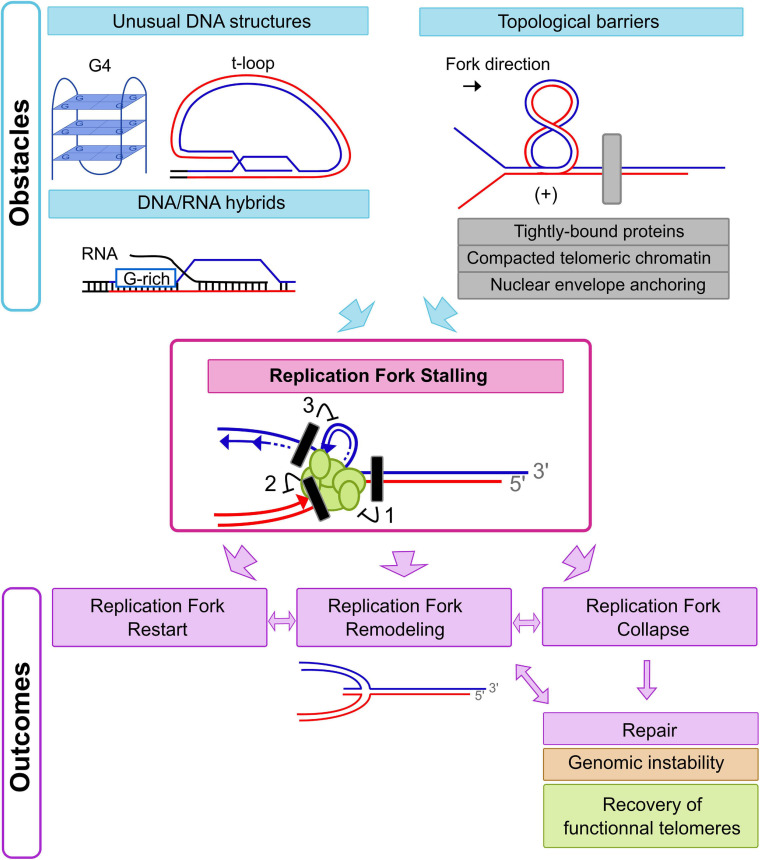
Initiation and outcomes of Replication Fork Stalling at chromosomal ends. Replication forks could stall just upstream to or on telomeric repeat tracts due to different obstacles. Hampering of replication fork progression may be caused by an incapacity of DNA unwinding by replicative helicases (block 1), a situation expected in the context of topological barriers (gray rectangle on the figure). Tightly bound proteins, compacted telomeric chromatin, and nuclear envelope anchoring are strong topological barriers at chromosomal ends. In humans, the unusual DNA structure of the t-loop could also induce a topological stress in front of the replication fork. At least two other situations could induce replication fork stalling with lesions inhibiting only leading strand synthesis (block 2) or lagging strand synthesis (block 3). Given that G4s could be formed on the G-rich strand (blue line) during lagging strand synthesis, a lagging strand specific defect could be expected with this kind of replication stress. In contrast, t-loops or DNA/RNA hybrids could lead to leading strand synthesis defects. Depending on the kind of replication stress encountered, there are various pathways to deal with the consequences of a stalled replication fork. Replication restart can occur by alleviation of the replication stress and repriming events. Replication fork remodeling with fork reversal could also follow replication fork stalling. In addition, complete collapse of the replication fork could occur, resulting in DSBs or one-sided DSBs that initiate appropriate or inappropriate repair pathways.

Replication stress can be defined by the transient slowing or arrest of replication fork progression (Zeman and Cimprich, 2014). At chromosomal ends, slow replication fork progression or fork pausing has been observed in budding and fission yeasts (Ivessa et al., 2002; Makovets et al., 2004; Miller et al., 2006), as well in higher eukaryotes (Verdun and Karlseder, 2006). Consequently, telomeres are part of the so-called “hard-to-replicate regions” and an endogenous source of replication stress. Many obstacles can slow or arrest replication fork progression including DNA lesions, unusual DNA structures, collisions with transcriptional machinery or RNA-DNA hybrids [Figure 2, top; (Zeman and Cimprich, 2014)]. The impact of telomeric DNA lesions on replication fork progression such as oxidative DNA damage will not be addressed in this review (Barnes et al., 2019). Here, we aim to focus on and describe main sources of replication stress at chromosomal ends imposed specifically by telomeric chromatin (Figure 2).

The nature of the sequences of the telomeric repeats render them prone to adopt unusual DNA structures. Indeed, telomeres are composed of G-rich repetitive DNA that can form G-quadruplexes (G4s) or other non-B DNA structures *in vitro* (Tran et al., 2011; Jurikova et al., 2020). G-quadruplexes are formed by stacking of 2 or more G-tetrads (a planar array formed by 4 guanines) (Figure 2, top left panel). Multiple G4-forming sequences have been identified in genomes potentially yielding quadruplex structures with different topologies and stabilities *in vitro* (Todd et al., 2005; Burge et al., 2006; Capra et al., 2010). Whereas certain indirect evidence tends to confirm a presence of unusual DNA such as G4s *in vivo* at telomeres [reviewed in Bochman et al. (2012)], direct evidence of their presence (or absence) is technically difficult to obtain. Using *in vitro* conditions close to physiological states, it has been shown than ssDNA made up of human telomeric repeats (5′-TTAGGG-3′) folds into stable anti-parallel G4s, whereas G4s were unfolded when the complementary strand was present (Kreig et al., 2015). Hence, in terms of thermodynamics, folding of G4s formed by human telomeric repeats is unfavored as compared to dsDNA and favored compared to ssDNA (Lane et al., 2008). Consequently, DNA unwinding of the pre-existing dsDNA in front of the replisome should not be impaired by the presence of G4s. However, telomeric G-rich ssDNA is exposed behind the replisome and is the template for lagging strand replication, in essence providing for a temporal window for possible G4 folding. Those structures then could block DNA synthesis by DNA polymerase δ on the lagging strand (Woodford et al., 1994; Figure 2, replication fork stalling block 3). Additionally, in higher eukaryotes, the terminal telomeric single strand DNA extension invades telomeric duplex DNA forming a particular DNA structure called the t-loop (Griffith et al., 1999; Doksani et al., 2013; Figure 2, top left panel). This structure protects the chromosomal end from being processed as DSB and must be dismantled before the replication fork arrives in order to avoid replication stress. It should be noted that at telomeres from single cell eukaryotes such as *S. cerevisiae*, it is very unlikely t-loops are present because the single strand extensions observed in this model organism are extremely short (Larrivée et al., 2004; Wellinger and Zakian, 2012; Soudet et al., 2014).

In addition to the specific telomeric DNA structure, transcription from subtelomeric and telomeric areas and the presence of RNA-DNA hybrids could hamper fork progression. Indeed, different species of non-coding RNAs produced from subtelomeric and telomeric areas in yeasts and vertebrates, including humans, have been described (Azzalin et al., 2007; Luke et al., 2008; Schoeftner and Blasco, 2008; Bah et al., 2012; Greenwood and Cooper, 2012). Of the different subtelomeric and telomeric non-coding RNA species identified so far, telomeric repeat-containing RNAs (TERRAs) are arguably the most intensively studied. This is due to their conserved presence in many species and their role in telomere biology [reviewed in (Azzalin and Lingner, 2015)]. Transcribed by RNA polymerase II in a cell-cycle regulated fashion, these heterogenous-sized RNAs contain subtelomeric sequences and telomeric G-rich repeats (Azzalin et al., 2007; Luke et al., 2008; Porro et al., 2010; Graf et al., 2017). TERRA’s association to telomeric chromatin is most likely through formation of telomeric R-loops (Balk et al., 2013; Pfeiffer et al., 2013; Arora et al., 2014; Figure 2, left panel). In budding yeast, the removal of TERRA R-loops is cell-cycle regulated and occurs in late S-phase, coinciding with telomere replication (Graf et al., 2017). Conceptually, this finding is consistent with removal of telomeric R-loops before replication fork arrival, limiting potential replication stress induced by telomeric RNA-DNA hybrids.

The chromatin at chromosomal ends encompasses several particularities such as heterochromatin or a heterochromatin-like organization and binding of shelterin or shelterin-like complexes (Figure 1B). Heterochromatin has initially been described as chromosomal regions staying condensed through the cell cycle. Nowadays, the definition of heterochromatin has become more a question of the presence, or absence, of specific post-translational modifications on histones such as H3K9me3 and chromatin association of HP1 (Nishibuchi and Déjardin, 2017). Whereas telomeres and subtelomeres in humans have been considered to be organized as constitutive heterochromatin, recent data challenge this view as in most human cell lines an enrichment of H3K9me3 at telomeres could not be found (Cubiles et al., 2018; Gauchier et al., 2019). In budding yeast, a few loci, including telomeres, exhibit a heterochromatin-like organization characterized in this model organism by chromatin enriched with the SIR complex (Sir2, Sir3, and Sir4) (Ellahi et al., 2015). However, SIR-bound chromatin at chromosomal ends is limited to telomeric chromatin and subtelomeric repetitive X elements (Ellahi et al., 2015). Moreover, this particular chromatin seems to play little role in replication fork arrest observed upstream of the compacted telomeric chromatin (Makovets et al., 2004). This is consistent with observations that the telomeric chromatin is devoid of nucleosomes and seems compacted even in the absence of SIR proteins (Wright et al., 1992; Pasquier and Wellinger, 2020). Indeed, replication fork arrest at chromosomal ends appears to depend on binding of Rap1, the major telomeric dsDNA binding protein in budding yeast (Makovets et al., 2004). This tightly associating DNA-binding protein consequently could be a source of telomeric replication stress (reviewed in (Dalgaard et al., 2011; Figure 2, top right panel). Binding of Rap1 to DNA relies on a MYB-like domain and impacts the topology of DNA (Müller et al., 1994). Interestingly, TRF1 and TRF2, the DNA-binding proteins of the human shelterin complex, also bind telomeric DNA via a MYB-like domain called the telobox (Chong et al., 1995; Bilaud et al., 1997; Broccoli et al., 1997). *In vitro*, DNA-bound TRF1 and TRF2 block replication fork progression (Ohki and Ishikawa, 2004) and TRF2 impacts telomeric DNA topology (Amiard et al., 2007; Poulet et al., 2012). Moreover, TRF2 overexpression leads to increased replication fork stalling on telomeric repeats (Nera et al., 2015). Telomeric dsDNA binding protein Taz1 is the functional homolog of the TRF proteins in *S. pombe* and also bears a C-terminal Myb domain (Cooper et al., 1997; Deng et al., 2015). This suggests that a tight binding of telomeric repeats by particular proteins is evolutionarily conserved. While this arrangement could hamper replication fork progression, there may be benefits to it as well, as deletion of TRF1 in mammals and Taz1 in *S. pombe* leads to frequent fork stalling (Miller et al., 2006; Sfeir et al., 2009) (see section “Multiple Pathways Helping Replication Fork Passage Through Chromatinized Telomeres” below for further discussion).

Some sources of telomeric replication stress described here involve slowing or arrest of replication fork progression by a topological stress in front of the replication fork (Figure 2, top right panel). Indeed, unwinding parental DNA duplexes by replicative helicases leads to accumulation of positive helical stress in front of a replication fork. If not resolved, these can further inhibit replication fork progression (Schalbetter et al., 2015; Keszthelyi et al., 2016; Shyian et al., 2019; Larcher and Pasero, 2020; Minchell et al., 2020). Unusual DNA structures like the telomeric t-loop in mammals, or the evolutionarily conserved compacted telomeric chromatin are expected to inhibit free DNA rotation and consequently to be a strong topological barrier (Kegel et al., 2011) [(discussed in this review (Giraud-Panis et al., 2013)]. Anchoring of telomeres at the nuclear envelope, a relatively well evolutionarily conserved feature of telomeric chromatin, is another potential source of topological stress at telomeres during replication (Chikashige et al., 2010; Taddei et al., 2010; Burla et al., 2016; Whalen and Freudenreich, 2020). Nonetheless, the cell cycle phase dependent regulation of telomere anchoring to the nuclear envelope disfavors this possibility, notably in human cells (Crabbe et al., 2012). While in budding yeast a delocalization of telomeres from the nuclear periphery appears to correlate with replication timing, direct evidence of telomere anchoring to the nuclear envelope during telomere replication is lacking (Hediger et al., 2002; Ebrahimi and Donaldson, 2008).

### Outcomes of Replication Fork Stalling at Chromosomal Ends

Knowing that the inherent characteristics of telomeres in yeasts as well as in vertebrate cells are a source for endogenous replication stress and therefore conserved features, the question arises of whether slowing replication fork progression at chromosomal ends could be somehow beneficial to complete chromosomal replication. It is clear that without appropriate DNA replication restart or fork protection, the outcome of telomeric fork stalling could be detrimental to cell survival and lead to genomic instability. At most genomic locations, fork stalling can be compensated by a convergent replication fork that arrives at the specific locus from the other side. For terminal telomeric repeat DNA, there is no evidence of a convergent replication fork able to rescue stalled forks in yeast model organisms, but there is growing evidence of possible replication origin firing inside mouse and human telomeres (Sfeir et al., 2009; Drosopoulos et al., 2020, 2012). Indeed, origin firing within telomeres, favored by direct interaction of TRF2 with ORC in humans, has been detected in mouse and human cells by a method called single molecule analysis of replicated DNA (SMARD) (Sfeir et al., 2009; Drosopoulos et al., 2020, 2012). While this technique is not applicable to yeast model organisms because of their very short telomeric repeat tracts, functional studies have shown that even if such origins existed, their efficacy is too low to maintain very short artificial chromosomes (Wellinger and Zakian, 1989). Moreover, initiation within telomeres seems to be a very rare event at human chromosomal ends, suggesting that even in human cells, telomeres are mainly replicated by replication forks originating in subtelomeric areas and moving from the centromeres toward telomeres (Drosopoulos et al., 2012). Therefore, restart of DNA replication at telomeres would mainly be dependent on conservation of fork integrity and the ability of the cells to alleviate the replication stress source (Figure 2, bottom panel).

In some instances, fork remodeling is observed under replication stress conditions. Specifically, re-annealing of the parental DNAs and annealing of the nascent strands, thereby forming a four-way junction, may occur. This mechanism is called replication fork reversal and previously was considered a pathological threat potentially leading to genomic instability. However, fork reversal is now thought to be beneficial under some circumstances (Neelsen and Lopes, 2015; Figure 2, bottom panel). Indeed, by promoting the DNA damage tolerance pathway or by limiting fork uncoupling and ssDNA accumulation, replication fork reversal could promote proper DNA replication (Neelsen and Lopes, 2015). However, when fork integrity is not maintained following stalling or when replication stress cannot be alleviated or bypassed, the replication fork would collapse (Figure 2, bottom panel). Replication fork collapse may be defined by the incapacity to resume DNA synthesis at the fork (Zeman and Cimprich, 2014). If such collapses are too frequent and persist into mitosis, the presence of under-replicated DNA regions will lead to formation of anaphase bridges, DSBs and ultimately chromosomal segregation defects, major threats to genomic stability (Bizard and Hickson, 2018; Stroik and Hendrickson, 2020a). Inappropriate repair of the DSBs by NHEJ or Alt-NHEJ pathways leading to sister chromatid fusion or chromosomal end-to-end fusions are possible outcomes, yet again resulting in genome instability (Rai et al., 2010). On the other hand, in telomeric repeats, a one-sided DSB would be generated at sites of stalled replication forks by the action of nucleases. On such a site, extension by telomerase is a way to avoid the catastrophic telomere shortening and possible deleterious outcomes of a telomeric replication fork collapse. In addition, the action of homology-dependent recombination (HDR) repair pathways could also allow recovery of functional telomeres after telomeric fork collapse [see section “Alternative Lengthening of Telomeres”; for review, (Stroik and Hendrickson, 2020b)].

In mammalian cells, several telomere phenotypes have been linked to telomeric replication defects and include telomere loss and sister telomere exchange or telomere fragility. These phenotypes are based on FISH (Fluorescence *in situ* Hybridization) experiments done on metaphase chromosomes [reviewed in (Cherdyntseva and Gagos, 2020)]. Telomere loss or sister telomere loss refers to absence of telomeres or the repeat array has become too short to be visualized by FISH. As mentioned above, abrupt telomere loss could be a consequence of telomeric fork collapse followed by its nucleolytic cleavage. Telomere fragility is characterized by broken or decondensed telomeres visible as multiple telomeric signals by FISH (Sfeir et al., 2009). Precise molecular mechanisms leading to this latter phenotype still are only partially understood. However, recently it has been shown that DSB formation and the BIR (Break-Induced Replication) repair pathway were involved in formation of fragile telomeres (Yang et al., 2020). Telomeric sister chromatid exchange could be detected by CO-FISH (Chromosome Orientation-FISH), a strand-specific variant of FISH and this phenotype is associated with telomeric replication defects (Cherdyntseva and Gagos, 2020). Finally, detection of Mitotic DNA synthesis (MiDAS) at telomeres in mammalian cells is also thought to be a consequence of telomeric fork progression defects (Özer et al., 2018).

### Multiple Pathways Helping Replication Fork Passage Through Chromatinized Telomeres

Many factors that are involved in the completion of telomere replication by conventional machinery have been identified (Higa et al., 2017; Maestroni et al., 2017). These factors aid in “conventional” telomere replication by not only alleviating sources of replication stress, but by allowing fork protection, fork remodeling and fork repair as well. From the various factors involved in this process, we would like to emphasize evolutionarily conserved pathways such as diverse helicases, the Fork Protection Complex (FPC), topoisomerases and proteins involved in HDR.

Multiple helicases are involved in telomeric replication by conventional replication machinery, likely acting to alleviate some sources of replication stress or promoting fork remodeling and repair. In budding yeast, the 5′-3′ DNA helicase Rrm3 helps replication fork progression through non-nucleosomal replication fork barriers, notably at telomeres (Ivessa et al., 2003, 2002). In humans, it has been demonstrated that members of RecQ-like helicases such as BLM and WRN, and RTEL1 from the iron-sulfur–containing DNA helicase family are required for proper telomere replication (Crabbe et al., 2004; Hao et al., 2004; Zimmermann et al., 2014). DNA helicases can be recruited to chromosomal ends by protein-protein interactions with replication fork components [e.g., Rrm3 (Azvolinsky et al., 2006)] or directly with shelterin subunits [e.g., BLM, WRN, and RTEL1 (Opresko et al., 2002; Lillard-Wetherell et al., 2004; Machwe et al., 2004; Zimmermann et al., 2014)]. The interplay between shelterin components TRF1 and TRF2 to recruit DNA helicases appears complex and highly regulated, notably by post-translational modifications [for review, see (Cicconi and Chang, 2020)]. For example, TRF2 recruits the BUB1-BUB3 complex at telomeres in S-phase, leading to phosphorylation of TRF1 (Li et al., 2018). TRF1 phosphorylated by BUB1 allows recruitment of the BLM helicase, favoring complete telomere replication (Li et al., 2018). Moreover, whereas a phospho-switch on TRF2 allows RTEL1 telomere recruitment in S-phase, probably in order to unwind the t-loop, binding of RTEL1 to PCNA is also implicated in “conventional” telomeric replication of the lagging strand (Vannier et al., 2012; Margalef et al., 2018; Sarek et al., 2019, 2016). Recruitment of DNA helicases through direct interaction with TRF1 and TRF2 at least in part explains the known beneficial roles of TRF1 and TRF2 in telomeric replication fork progression *in vivo* (Sfeir et al., 2009). Interestingly, Taz1, the *S. pombe* ortholog of TRF1 and TRF2, is also necessary for faithful telomere replication (Miller et al., 2006). Tbf1, the budding yeast ortholog of TRF1/TRF2 bound at subtelomere-telomere junctions, impacts telomere length homeostasis (Berthiau et al., 2006). However, a possible implication of Tbf1 in replication of chromosomal ends has yet to be addressed experimentally.

In addition to helicases helping the replication machinery pass though protein-bound telomeres, topoisomerases play a role in telomere replication. Indeed, TOPOIIα in concert with TRF2 and the nuclease Apollo is involved in proper telomere replication in humans (Ye et al., 2010). TOPOIIα prevents telomere fragility and likely is recruited to telomeres through its interaction with TRF1 (D’Alcontres et al., 2014). Similarly, fission yeast TopoII also appears to be implicated in resolution of telomere replication intermediates (Germe et al., 2009). In addition, it has been proposed that the BLM helicase is associated with telomeres in a cell-cycle regulated manner and recruits TOPOIIIα-RMI1-RMI2 (BTR complex) to allow proper chromosome segregation by limiting anaphase bridge formation (Barefield and Karlseder, 2012). Another complex that appears important for conventional telomere replication from *S. pombe* to humans is the Fork Protection Complex (FPC, composed of Timeless, Tipin, And1, and Claspin proteins in humans) (Leman et al., 2012; Gadaleta et al., 2016). The FPC coordinates DNA-replication checkpoint activation and cohesin establishment at replication forks [reviewed in (Leman and Noguchi, 2012)]. The Timeless protein associates with the shelterin subunit TRF1 and Timeless-depleted cells show decreased telomere length (Leman et al., 2012). The requirement of the FPC for proper telomeric replication again highlights the occurrence of frequent fork stalling at chromosomal ends. In budding yeast, Tof1, the homolog of human Timeless, also has numerous roles in regulation of replication fork stability as well as in action of topoisomerases ahead of the fork (Schalbetter et al., 2015; Shyian et al., 2019; Larcher and Pasero, 2020; Westhorpe et al., 2020). In addition, Tof1-depleted cells show more heterogeneity in telomere size than WT cells (Grandin and Charbonneau, 2007).

Whereas most helicases mentioned previously have known roles in HDR, numerous other proteins involved in HDR are necessary to complete “conventional” telomere replication. In mammals, the ATM and ATR kinases are recruited to chromosomal ends and are required for proper telomere replication (Verdun and Karlseder, 2006; McNees et al., 2010; Pennarun et al., 2010). ATM and ATR are two major kinases orchestrating DNA Damage Response (DDR) pathways to preserve genome integrity [reviewed in (Maréchal and Zou, 2013)]. The ATM kinase (Tel1 in budding and fission yeasts) is mainly activated by DSBs, whereas the ATR kinase (Mec1 in budding yeast, Rad3 in fission yeast) is mainly activated by RPA-coated single strand DNA [reviewed in (Maréchal and Zou, 2013)]. Interestingly, in budding yeast, despite having all telomerase holoenzyme components, *tel1*Δ *mec1*Δ cells behave like telomerase-negative cells, exhibiting telomere shortening and senescence (Ritchie et al., 1999). Moreover, fission yeast devoid of the two major DDR kinases also behave like telomerase-negative cells (Naito et al., 1998; Nakamura et al., 2002). These results demonstrate that activity of DDR kinases is necessary to properly maintain telomeric ends, likely by allowing appropriate processing of telomeres, i.e., post-replicative end processing and telomerase activation and/or recruitment [more details on the link between DDR kinases and appropriate processing of telomeres can be found in these reviews (Doksani and de Lange, 2014; Vasianovich et al., 2019)]. These results suggest also that recognition of telomeres as DNA damage (in a controlled manner) is a prerequisite to genome stability. In this context, replication stress at telomeres could be beneficial by allowing recruitment of major DDR kinases in a narrow temporal window. However, whereas deletion of *TEL1* in budding yeast leads to a pronounced short telomere phenotype, bulk telomere length is only slightly affected in *mec1*Δ *sml1*Δ cells (Craven et al., 2002). In contrast, in fission yeast, no obvious telomere phenotype is observed in absence of *TEL1*, but a pronounced short telomere phenotype is observed in the absence of *RAD3* (Nakamura et al., 2002). These results suggest that Tel1 is the DDR kinase predominantly recruited and activated at telomeres in budding yeast whereas Rad3 fills this role in fission yeast. Given the differences in recruitment of ATM homologs (Tel1) and ATR homologs (Mec1 in budding yeast, Rad3 in fission yeast), these results suggest that the main telomeric DNA substrates sensed as DNA damage during replication from these model organisms are different. In budding yeast, knowing that telomeric DNA substrates from post-conventional replication resemble a DSB, i.e., blunt ends from leading strand synthesis, Tel1 could be recruited and activated at the leading strand. However, in absence of Tel1, DDR kinase activity by telomeric Mec1 recruitment is sufficient to maintain enough telomerase activity at chromosomal ends to avoid senescence. Mec1 recruitment could be achieved through exposure of RPA-coated single strand non-telomeric DNA following resection (single strand telomeric DNA is very likely coated by Cdc13 in budding yeast, see below). Conversely, given that in fission yeast, lagging strand synthesis is delayed compared to leading strand synthesis at chromosomal ends (Moser and Nakamura, 2009), the resulting ssDNA accumulation could lead to a preferential recruitment of Rad3 for this model organism, contrary to what happens in budding yeast. This model was supported by experiments showing an association of RPA with telomeres that coincides with the arrival of replication fork. Furthermore, a specific RPA mutant leads to issues in telomeric lagging strand replication and/or telomerase extension in fission yeast (Faure et al., 2010; Luciano et al., 2012; Chang et al., 2013; Audry et al., 2015). Whereas RPA association to telomeres during replication also seems to occur in budding yeast, the specific role of RPA in this system is less defined (Luciano et al., 2012; Markiewicz-Potoczny et al., 2018).

### Telomere Replication Without Active Telomerase

In budding and fission yeasts, expression of all required telomerase subunits is constitutive. Unlike in these unicellular eukaryotes, telomerase is not active in the majority of human somatic cells after the embryonic stage and these cells have a very limited capacity of lengthening short telomeres (Wright et al., 1996). Without active telomerase, the natural shortening of telomeres that occurs at each replicative division in human somatic cells is an important mechanism for preventing cancerous cell transformation. Indeed, when a certain lower threshold for telomeric repeat length is reached, telomeres become dysfunctional, triggering a terminal cell cycle arrest that leads to replicative senescence. Therefore, normal telomere attrition during DNA replication acts as a barrier to unlimited cell divisions. Abnormalities in telomere replication promote instability with various potential outcomes: programmed senescence, cell death, or even more deleterious genome instability leading to oncogenic transformation.

#### Telomerase-Negative Yeast Cells

Yeasts are excellent model organisms to study replicative senescence due to the ability to genetically manipulate telomerase expression. Although telomerase is constitutively expressed in budding yeast, it can be inactivated through deletions of the genes coding for critical components of the holoenzyme (Lundblad and Szostak, 1989; Lundblad and Blackburn, 1993). The ensuing absence of telomerase eventually will lead to critically short telomeres, just as in humans. This occurs either by gradual telomere shortening of 3–5 bp per population doubling or sudden major telomeric repeat loss events (Marcand et al., 1999). When this crisis point occurs, cells enter a Mec1-dependent irreversible G2/M arrest (Chen et al., 2001). A very small subset of cells evade this permanent arrest by using recombination-based mechanisms to regenerate telomeres, thus forming “survivors” (Lundblad and Blackburn, 1993). Like in budding yeast, absence of the telomerase protein subunits or the RNA template results in replicative senescence in *S. pombe* (Nakamura et al., 1998, 1997; Webb and Zakian, 2008). A small number of these cells also form survivors, although unlike budding yeast, the majority of survivors are formed by chromosome circularization and only a small subset by recombination (Nakamura et al., 1998). This difference is most likely related to the lesser number of chromosomes in *S. pombe* (3) compared to *S. cerevisiae* (16), as genetically engineered single chromosome budding yeast was able to produce survivors with circularized chromosomes (Wu et al., 2020). Interestingly, in fission yeast a new survivor type termed HAATI-survivors has been described (heterochromatin amplification-mediated and telomerase-independent) (Jain et al., 2010). In these HAATI-survivors, chromosome linearity did not rely on the presence of canonical telomeres, based on telomeric repeat DNA, at chromosomal ends, but instead on the presence of non-telomeric heterochromatin (Jain et al., 2010; Begnis et al., 2018).

Further studies in budding yeast were the first to lead to the discovery of genetic requirements of telomerase-independent mechanisms of telomere maintenance, termed ALT for Alternative Lengthening of Telomeres. Recently, the overall rate of survivor frequency was determined as 2 × 10^–5^ (Kockler et al., 2021). Conventionally, it was believed that in *S. cerevisiae* two types of survivors are formed: type I arise through amplification of the subtelomeric Y’ sequences and type II are formed by amplification of the terminal telomeric repeats, with obligate genetic factors varying between the two types (Lundblad and Blackburn, 1993; Le et al., 1999; Teng and Zakian, 1999). Regardless of type, survivor formation is dependent on Rad52 for homologous recombination (HR) and Polδ subunit Pol32 for break-induced-replication (BIR) (Lundblad and Blackburn, 1993; Lydeard et al., 2007). BIR is used to repair one-ended DSBs and arrested replication forks through strand invasion of a DSB into a homologous donor sequence which is used as a template for unidirectional replication [reviewed in (Kramara et al., 2018)]. Due to the terminal position of telomeres, replication-induced telomeric breaks are essentially single-ended DSBs that cannot be rescued by a converging replication fork, thus in the absence of telomerase require BIR for repair (Lydeard et al., 2007). However, recent work using a novel approach of populational and molecular genetics combined with ultra-long sequencing challenges this long-established paradigm of two independent survivor pathways: the RAD51-dependent pathway generating type I survivors, and the RAD59-dependent pathway generating type II survivors (Kockler et al., 2021). Instead, it is proposed that ALT occurs through a unified pathway with two sequential steps, formation of ALT precursors using *RAD51*-mediated strand invasion followed by their maturation into ALT survivors via a *RAD59*-dependent pathway. Consistent with this proposal, analyses of ultra-long sequencing of chromosome terminal sequences derived from survivor cells revealed hybrid sequences containing features attributed to both types of survivors (Kockler et al., 2021).

#### Inactivation of Telomerase in Yeasts Points to Frequent Telomere Replication Stress

Despite the gradual telomere shortening observed in telomerase-negative budding yeast, in such cultures the vast majority of cells most likely arrest due to critically short telomere(s) that arose via a single major loss event of telomeric repeats. It is thought that this event is triggered by stresses encountered during DNA replication and the resulting single critically short telomere is enough to cause growth arrest (Abdallah et al., 2009; Khadaroo et al., 2009; Xu et al., 2013). Consistent with this, telomerase inactivation rapidly exposes problems associated with telomeric replication stress, even before bulk telomere shortening reaches a critical point (Ijpma and Greider, 2003; Khadaroo et al., 2009; Jay et al., 2016; Xu and Teixeira, 2019). Observation of the dynamics of individual telomerase-negative cell lineages very early after inactivation of telomerase has recently been made possible by using a microfluidics device coupled with an inducible telomerase-null mutant. Results from experiments using this system confirm highly heterogenous cell cycle durations (even in cells of the same lineage) and transient cell cycle arrests well before bulk telomere shortening-induced arrest (Xie et al., 2015; Xu et al., 2015).

The relationship between replication stress and telomere recombination in telomerase-negative yeast indicates that telomerase may play an important role in repair of replication stress-induced damage at telomeres. In the absence of telomerase, multiple repair mechanisms involving checkpoint mediators, recombination factors, DNA damage adaptors, and post-replication repair are required for telomere healing [reviewed in (Simon M. N. et al., 2016)]. A variety of factors in these different pathways have been identified as delaying senescence, as upon their removal the onset of senescence is accelerated [reviewed in (Simon M. N. et al., 2016; Xu and Teixeira, 2019)]. Further supporting the idea that replication stress is unmasked in the absence of telomerase, elevation of dNTP pools (facilitating replication) alleviates the early senescence seen in the absence of DNA damage adaptors (Jay et al., 2016). The onset of senescence can also be delayed by short terminal TG_1__–__3_ repeats of the G-rich overhang engaging in BIR with interstitial telomeric sequences (ITSs). These sequences are located in the subtelomeric region and can be used in order to repair a broken telomere by non-reciprocal translocation mechanisms (Churikov et al., 2014). How the G-rich ssDNA overhang pairs with dsDNA ITSs is not fully understood, however it is hypothesized that unwinding of DNA during replication of the subtelomeric region may facilitate initiation of recombination (Churikov et al., 2014).

#### Alternative Lengthening of Telomeres

As previously mentioned, most human somatic cells are telomerase-inactive, thus have no inherent mechanism to maintain telomere length, losing telomeric repeats at each cell division. However, as observed in telomerase-negative yeast cells, certain cells can escape replicative senescence through either the re-expression of the lacking telomerase subunits or homology-directed repair (HDR) mechanisms, thus leading to an unlimited proliferative potential [reviewed in (Shay, 2016)]. Telomerase-independent immortalization through BIR-mediated homology-directed repair (HDR), similar to the Rad52- and Pol32-dependent mechanisms seen in survivor formation in *S. cerevisiae*, is observed in 10–15% of human cancers, and these are known as ALT cells (Alternative Lengthening of Telomeres) (Bryan et al., 1995, 1997). ALT cells possess several prominent features, notably extrachromosomal telomeric DNA in the form of C-circles and G-circles, increased telomeric-repeat length heterogeneity, increased formation of ALT-associated PML bodies (APBs), telomere dysfunction-induced foci (TIFs), and increased frequency of telomere sister chromatin exchange [reviewed in (Sobinoff and Pickett, 2020)]. Like in yeast, BIR-mediated ALT cell formation also requires DNA polymerase δ subunits (POLD3/4) (Costantino et al., 2014; Dilley et al., 2016; Roumelioti et al., 2016). RAD52 can be implicated, however recent data support a RAD52-independent ALT pathway involved in the formation of C-circles (Min et al., 2017, 2019; Zhang et al., 2019). As thoroughly discussed in recent reviews, both intrinsic and extrinsic DNA replication stress at mammalian telomeres may be important ALT activators, although triggers of this stress remain to be fully elucidated [reviewed in (Domingues-Silva et al., 2019; Sobinoff and Pickett, 2020; Stroik and Hendrickson, 2020b; Zhang and Zou, 2020)]. Thus, proteins involved in the response to and resolution of replication stress are critical in suppressing the formation of ALT cells, and by extension, the potential proliferative potential of a subset of cancer cells. Notably, recent work from multiple labs has highlighted the importance of the Fanconi Anemia (FA) protein FANCM in the suppression of ALT, likely through alleviating telomeric replication stress and damage by regulating BLM helicase activity and preventing telomeric R-loop accumulation (Pan et al., 2017; Lu et al., 2019; Silva et al., 2019), [reviewed in (Domingues-Silva et al., 2019)].

An interesting hypothesis proposes that telomerase efficiently repairs replication stress damage at telomeres either by directly elongating the accidentally broken telomere or by acting on the newly formed end exposed at a regressed replication fork (Noël and Wellinger, 2011; Simon M. N. et al., 2016). Thus, without telomerase, processing of a stalled fork or accidental breakage results in telomeres that are very short and recombinogenic. Consistent with this, telomerase can act as a repair enzyme at broken telomeres in *S. pombe* by binding to 3′ G-rich ssDNA created by reversed or broken replication forks, thereby recuperating telomere replication and protecting telomeres from inappropriate HDR (Matmati et al., 2020). In the absence of telomerase, fork restart was again dependent on HDR factors such as Rad51 and the MRN complex. On one hand, mammalian cells without telomerase, like yeast, either experience more telomere replication stress or are more sensitive to it, rendering it more readily detectable by experiments. As point in case, in cells that have achieved immortalization through ALT, multiple factors associated with replication stress are constitutively associated with these ALT telomeres (Arora et al., 2014; Pan et al., 2017). Thus, given that replication stress hinders cell cycle progression through activation of DNA damage checkpoints, mechanisms that alleviate ALT-specific Telomere Replication Stress (ATRS) must also be continually active to maintain ALT cell proliferation [reviewed in (Domingues-Silva et al., 2019)]. On the other hand, telomerase itself seems, at least in the context of some telomere replication defects, to become an issue of replication stress. For example, in RTEL1-deficient mouse cells, telomerase prevented replication fork restart by inappropriately binding to and stabilizing reversed forks (Margalef et al., 2018). Currently, there is a dearth of knowledge on replication intermediates and repair mechanisms at collapsed forks during human telomeric replication, thus making it a very interesting avenue of future research.

### Re-establishment of Functional Telomeres: Regeneration of the 3′ Overhang and the CST Complex

The process of semi-conservative DNA replication through the bulk of the telomeric tract leads to the DNA-end replication problem, wherein nucleolytic processing of the leading strand in yeast and both strands in mammals is required to regenerate the obligatory 3′ overhang (Soudet et al., 2014; Wu et al., 2012; Figure 1C). At lagging strand telomeres, removal of the last Okazaki fragment is thought to generate the appropriate 3′ ssDNA structure. Conversely, after passage of the replisome, leading strand telomeres are left as blunt ended replication intermediates necessitating 5′-to-3′ resection by nucleases such as Exo1 and Mre11, and subsequent C-strand fill-in for proper 3′ overhang regeneration (Lingner et al., 1995; Larrivée et al., 2004; Casteel et al., 2009; Soudet et al., 2014; Wellinger, 2014; Wu et al., 2012). Thus, the coordinated action of both telomerase and DNA polymerases is needed to fully replicate telomeres.

The heterotrimeric CST complex plays a critical role in forming the appropriate 3′ overhang structure and maintaining telomere homeostasis by facilitating telomere replication. The CST complex is highly conserved and is comprised of Cdc13-Stn1-Ten1 in *S. cerevisiae* and CTC1-STN1-TEN1 in mammals (Miyake et al., 2009; Price et al., 2010; Lue, 2018). In *S. pombe*, a Cdc13/CTC1 homolog is lacking (or undiscovered), and the 3′ ssDNA overhang is bound by Pot1 (Baumann and Cech, 2001; Matmati et al., 2018). Nonetheless, like in other eukaryotes, the fission yeast Stn1 and Ten1 genes are critical for telomere function as their deletion results in telomere loss and chromosome circularization (Martín et al., 2007). In both budding yeast and mammals, CST loads on telomeric ssDNA and facilitates RNA priming and DNA synthesis by the DNA Polα-primase complex to fill in the C-strand (Lue et al., 2014; Mirman et al., 2018). However, in *S. pombe*, recruitment of DNA Polα-primase is facilitated by telomeric dsDNA binding proteins Taz1, Rap1, and Poz1 (Chang et al., 2013). Despite this, recent studies have affirmed the conserved role of fission yeast (C)ST in DNA replication, as it was determined that Stn1 is necessary for replication of subtelomeres and telomeres (Takikawa et al., 2017; Matmati et al., 2018).

Previous research has highlighted the functional and structural similarities between the CST complex and the similarly heterotrimeric replication protein A (RPA) complex (Gao et al., 2007; Gelinas et al., 2009; Sun et al., 2009; Giraud-Panis et al., 2010). However, multiple lines of evidence emphasize key differences between the two. Unlike RPA, CST exhibits preferential binding to telomeric G-strand ssDNA in a length-dependent manner (Chen et al., 2012; Bhattacharjee et al., 2016). There are significant differences in how the subunits of different complex members contribute to DNA binding and thus shape the overall architecture and stoichiometry of the complexes (Fan and Pavletich, 2012; Bhattacharjee et al., 2016). Recently conducted structural analyses have provided a wealth of information on the CST complex in both yeasts and humans. Ge et al. (2020) resolved the crystal structures of the Cdc13-ssDNA, Cdc13-Stn1, and Stn1-Ten1 complexes and built a model of a CST complex with a 2:2:2 stoichiometry. Although several structural features of the subunits are conserved among yeasts, there may still be differences in stoichiometry, as seen in *Candida glabrata*, which forms 2:4:2 or 2:6:2 complexes (Lue et al., 2013; Ge et al., 2020). Furthermore, cryo-electron microscopy was used to determine that human CST assembles on telomeric ssDNA as a decameric supercomplex (Lim et al., 2020). In addition to resolving the stoichiometry of human CST, this work unexpectedly demonstrated that human CTC1 has a greater structural similarity to RPA than the anticipated similarity to yeast Cdc13. Despite this however, the work further confirmed that overall molecular architectures and stoichiometries of the two complexes differ dramatically.

In *S. cerevisiae*, the CST complex promotes telomere homeostasis via several mechanisms. As the cell cycle progresses into late S phase and semiconservative DNA replication nears completion, removal of RNA primers at the lagging strand and resection at the leading strand produces 8-15 nt telomeric 3′ G-overhangs (Larrivée et al., 2004; Soudet et al., 2014). These overhangs are bound by Cdc13, which has a high specificity for the terminal telomeric G-strand and can bind the G-rich ssDNA either alone or as part of the CST complex (Grandin et al., 1997, 2001). Cdc13 facilitates recruitment of telomerase to telomeres through a Cdc13-Est1 interaction (Evans and Lundblad, 1999; Gao et al., 2007). This Cdc13-Est1 interaction is mutually exclusive of the Cdc13-(Stn1-Ten1) interaction which provides end protection to the terminal overhang (Nugent et al., 1996; Evans and Lundblad, 1999; Chen et al., 2018). Cdc13, Stn1, and Ten1 are all required for cell viability and telomere length regulation as loss-of-function mutations in each subunit result in the accumulation of excessive telomeric ssDNA and abnormal elongation of telomeres (Garvik et al., 1995; Grandin et al., 1997, 2001). However, Cdc13’s critical function in chromosome stability appears to be most likely in its DNA replication-dependent function and not its post-replication end capping role (Langston et al., 2020). Indeed, a Cdc13 defect disrupts replisome function, allowing 5′-DNA degradation and thus end-gaps on the lagging strand template, facilitating formation of an initial unstable chromosome. Consequently, Cdc13’s role in chromosome stability mostly likely comes from its role in lagging strand synthesis in S phase or in chromosome capping in G2/M as instability events are generated within a single cell cycle. This instability originates at the terminal telomeric repeats as frequencies of instability events remained unchanged when TG repeats were inserted internally (Langston et al., 2020). Interestingly, new data using a genetically engineered single chromosome yeast system further highlights a strong role for end-driven versus internal replication defects (Wu et al., 2020). Experiments performed after insertion of interstitial telomeric sequences (ITSs) into the linear single chromosome yeast suggest that the CST complex does not affect the replication of ITSs, thus underscoring the hypothesis that the function of the CST complex might be limited to the recruitment of Polα for lagging strand synthesis specifically on the terminal telomeric repeats. This idea does not completely exclude the possibility that the CST complex can initiate lagging strand synthesis on G-strands of ITSs. However, given that lagging strand can always be initiated distally from the ITS, the CST-mediated initiation on the ITS simply is not required, whereas it is absolutely required at the ends of the chromosomes. Consistent with these ideas, Cdc13 interacts with the lagging strand machinery during semi-conservative DNA replication (Faure et al., 2010). Indeed, the data show that CST is involved in recruitment of the DNA Pol α–primase complex to telomeric G-overhangs (Grossi et al., 2004). Recent crystal structure modeling data suggests that this is accomplished via the Cdc13OB1–Pol1 and Stn1–Pol12 interactions (Ge et al., 2020). Thus, CST could act as a telomeric specific complex allowing priming and DNA synthesis not only at 3′-termini but also repriming on the lagging strand in context of replication fork stalling at chromosomal ends. This proposed role of CST complex acting as a telomeric repriming complex was also proposed for the mammalian CST complex (Wu et al., 2012; Mirman et al., 2018). However, what happens between the eviction of telomerase and Polα–primase complex recruitment to the G-overhang remains to be elucidated. Ge et al. (2020) speculate on the coordination of these two processes through conformational changes induced by the CST complex, such as a switch from a Cdc13 DNA binding to CST DNA bound, thus further highlighting the necessity of temporal regulation of the extendible and non-extendible states of telomeres (Teixeira et al., 2004).

Human telomeres terminate in a 12–400 nt 3′ G-rich overhang that serves as a substrate for telomere elongation by telomerase (Makarov et al., 1997; McElligott and Wellinger, 1997; Zhao et al., 2008). Resection by ExoI and Apollo nucleases generates the leading end overhang and presence of the lagging end overhang is due to the arrest of the lagging strand synthesis ∼70–100 nt before the actual chromosome end in addition to nuclease-mediated resection (Chow et al., 2012; Wu et al., 2012). CST interaction with the TPP1-POT1 heterodimer regulates localization of the CST complex to telomeres (Wan et al., 2009; Wu et al., 2012). TPP1 stabilizes the telomere-telomerase interaction and the G-strand is elongated by around 60 nt (Sexton et al., 2014; Hockemeyer and Collins, 2015; Schmidt et al., 2016). In late S/G2 phase, the aforementioned CST-orchestrated C-strand fill in by DNA Pol α–primase terminates G-overhang maturation and prevents overextension of the G-strand by telomerase (Chen et al., 2012; Chen and Lingner, 2013). This CST-mediated priming for C-strand fill-in is as important as telomerase-mediated G-strand elongation in maintaining telomere length (Feng et al., 2017). When CTC1 is disrupted, the G-strand 3′ overhang elongates, while the C-strand decreases in length due to a deficiency in fill-in synthesis. Overall, this leads to gradual telomeric shortening similar to telomerase-negative cells (Feng et al., 2017). Moreover, when examining the role of CST in telomere hyper-resection, Mirman et al. (2018) found that the complex limits the formation of ssDNA at dysfunctional telomeres in a 53BP1-, RIF1-, and Shieldin-dependent manner. In addition to its role in generating proper 3′ overhangs, CST-mediated stimulation of the DNA Pol α–primase complex facilitates the fork restart mechanisms needed to compensate for fork stalling that inherently occurs during replication of the repetitive, G-rich telomeric DNA (Gu et al., 2012; Wang et al., 2012). In fact, STN1 or TEN1 depletion slows replication and leads to potential telomere loss and/or fragile telomeres in cells with long telomeres (Huang et al., 2012; Stewart et al., 2012; Kasbek et al., 2013). CTC1 and STN1 mutations have been implicated in the telomere-related Coats Plus syndrome and patients with CTC1 mutations exhibit telomere dysfunction that is consistent with telomeric DNA replication errors (Anderson et al., 2012; Chen et al., 2013; Simon A. J. et al., 2016). Importantly and in contrast to budding yeast, mammalian CST and the ST complex in *S. pombe* also appear to have extratelomeric functions in DNA replication and fork restart under conditions of replication stress that are outside the scope of this review (Price et al., 2010; Stewart et al., 2012; Wang et al., 2014, 2019; Lyu et al., 2021).

## Discussion

Our knowledge on proteins and mechanisms involved in helping the replication fork to reach chromosomal ends has greatly expanded in recent years. Human telomere replication appears to rely on significantly more factors than telomere replication in yeasts (see section “Multiple Pathways Helping Replication Fork Passage Through Chromatinized Telomeres”). The much longer repeat tracts as compared to yeasts could be the reason for an inherently increased potential for replication stress, therefore requiring more means for maintaining fork stability. However, we would like to propose an alternative view. An evolutionary key difference between yeasts and human cells resides in telomerase being constitutively expressed in yeast, whereas it is not expressed in most human cells. Thus, in yeasts, recovery from telomeric replication fork collapse could be achieved by telomerase action, as already observed in fission yeast (Matmati et al., 2020). This efficient means for recovery of replication fork collapse by telomerase may lead to an under-estimation of telomeric replication fork collapse frequency and proteins involved in solving this issue. Indeed, studies on telomerase-negative yeast cells suggest that, as in human somatic cells, efficient progression of replication forks at chromosomal ends relies on numerous additional proteins (see section “Telomere Replication Without Active Telomerase”). Research on telomere replication in telomerase-negative yeast cells therefore would enable greater understanding of fundamental aspects of recovery of replication fork stalling at chromosomal ends in human cells. Importantly, given the absence of active telomerase in these cells and therefore the inability to repair telomeric replication issues by telomerase, the factors/pathways involved in telomere replication by the conventional replication machinery gain crucial importance. Somewhat counterintuitively, recent work suggests that telomerase could in fact bind reversed telomeric replication forks in mouse cells deficient for RTEL1 and in this context induce catastrophic telomeric repeat loss (Margalef et al., 2018). While the absence of active telomerase in human somatic cells is an important mechanism to avoid uncontrolled proliferation, it has also been demonstrated that preventing excessive telomere elongation and regulating telomere length at a certain homeostatic level is important for maintaining the functional state of telomeres [reviewed in (Harrington and Pucci, 2018)]. It is therefore tempting to speculate that in certain multicellular organisms, repair of telomeric replication fork collapse by telomerase indeed has been evolutionarily counter-selected. In other words, many telomerase-independent pathways may have evolved to promote efficient replication fork recovery. This allows avoiding complications due to telomerase-mediated over elongation and at the same time limits the proliferation potential of the cells, curbing any potential runaway cell divisions that could lead to cancer. Further characterization of these mechanisms will help to gain a better understanding of the interplay of processes involved in maintaining genome stability.

Therefore, frequent fork stalling at telomeres in human cells, despite the known risks associated with them, may be somewhat beneficial as they allow local and transient action of major DDR kinases (ATM and ATR) at telomeres, required for post-replicative processing of ends and efficient engagement of repair activities. Hence, a deeper understanding of replication stress in somatic cells versus cancerous cells (telomerase-inactive vs -active) could be important in advancing development of new drugs in cancer biology (see section “Alternative Lengthening of Telomeres”).

## Author Contributions

EB and EP wrote the manuscript. RW amended and completed the manuscript. All authors contributed to the article and approved the submitted version.

## Conflict of Interest

The authors declare that the research was conducted in the absence of any commercial or financial relationships that could be construed as a potential conflict of interest.
